# Structural Predictive Model of Presenilin-2 Protein and Analysis of Structural Effects of Familial Alzheimer's Disease Mutations

**DOI:** 10.1155/2021/9542038

**Published:** 2021-11-29

**Authors:** Alejandro Soto-Ospina, Pedronel Araque Marín, Gabriel de Jesús Bedoya, Andrés Villegas Lanau

**Affiliations:** ^1^University of Antioquia, Faculty of Medicine, Group Molecular Genetics, Medellín, Colombia; ^2^University of Antioquia, Faculty of Medicine, Group Neuroscience of Antioquia, Medellín, Colombia; ^3^EIA University, School of Life Sciences, Research and Innovation in Chemistry Formulations Group, Envigado, Colombia

## Abstract

Alzheimer's disease manifests itself in brain tissue by neuronal death, due to aggregation of *β*-amyloid, produced by senile plaques, and hyperphosphorylation of the tau protein, which produces neurofibrillary tangles. One of the genetic markers of the disease is the gene that translates the presenilin-2 protein, which has mutations that favor the appearance of the disease and has no reported crystallographic structure. In view of this, protein modeling is performed using prediction and structural refinement tools followed by an energetic and stereochemical characterization for its validation. For the simulation, four reported mutations are chosen, which are Met239Ile, Met239Val, Ser130Leu, and Thr122Arg, all associated with various functional responses. From a theoretical analysis, a preliminary bioinformatic study is made to find the phosphorylation patterns in the protein and the hydropathic index according to the polarity and chemical environment. Molecular visualization was carried out with the Chimera 1.14 software, and the theoretical calculation with the hybrid quantum mechanics/molecular mechanics system from the semi-empirical method, with Spartan18 software and an AustinModel1 basis. These relationships allow for studying the system from a structural approach with the determination of small distance changes, potential surfaces, electrostatic maps, and angle changes, which favor the comparison between wild-type and mutant systems. With the results obtained, it is expected to complement experimental data reported in the literature from models that would allow us to understand the effects of the selected mutations.

## 1. Introduction

Neurodegenerative diseases are characterized by progressive damage to the nervous tissue. Alzheimer's disease (AD) is considered one of the most frequent diseases and here we contemplate multiple factors that trigger its onset [1, 2], such as genetic disposition due to variations present in the genes that translate proteins such as presenilin-1 (PS1), presenilin-2 (PS2), and amyloid precursor protein (APP) [3–5]. Similarly, the disease can be acquired due to sporadic disposition due to risk factors such as low physical and cognitive activity, toxicity due to bioaccumulation of metals in the brain such as copper and zinc, or due to a high-fat diet [6, 7].

The PS2 protein found in the coding region of chromosome 1 is part of the *γ*-secretase enzyme and is considered a protein homologous in function to the PS1 protein [8–11]. The active site of the PS2 protein is composed of two aspartic acids (D263 and D366) that have a protease action for approximately 93 substrates, as well as PS1 that has its active site in D257 and D385; hence, they are considered functional as they cleave the peptide bond in peptide or protein-type substrates [12–14].

At present, according to the databases consulted, such as Uniprot, EMBL, Human Protein Reference, and Protein Data Bank, there is no crystal of this protein. Taking this information into account, we make use of the protein analog to PS2, which is PS1 and has a large part of its structure solved, in order to generate the first model of this protein [11, 15, 16]. Computational models obtained through structure predictors are a great ally in obtaining hypothetical models to understand pathologies at the molecular level.

Alzheimer's disease conceives two phenotypes, which manifest themselves as late-onset Alzheimer's disease (LOAD) with an age of onset after 65 years, and early onset Alzheimer's disease (EOAD) with an age of onset under 65 years [17, 18]. Calcium ion is extremely important at the brain level, given that it is involved in many processes in neuronal metabolism, such as the coupling of the SNARE complex for the anchoring of vesicular neurotransmitter transporters and the release of such neurotransmitters. Furthermore, its role is key in the repolarization pertaining to the action potential mechanisms of neuronal synaptic signals [16, 19–21]. Therefore, it is sought to understand how mutations can affect the protease activity of the PS2 protein, and how some mutations can potentially affect or block cellular signal transduction due to their marked effect on phosphorylation [22]. To better understand this effect, it is necessary to define the concept of moonlighting, which encompasses the multitasking function of protein systems. These functions include receptor-type functions, activation of cell death, folding such as chaperones and chaperonins, ligases, proteases, oxidoreductases, regulation of transcription factors, and cell signaling in signal transduction [23–25]. In cell signaling, to induce a response, it is necessary for proteins to undergo a post-translational modification, such as phosphorylation, in which the alcohol functional group of the thyroxine, serine, and threonine amino acids can modify and a phosphate group added, which can produce conformational changes that trigger transduction signals for a specific cell function, depending on the biochemical activation pathway. These phosphorylations have been widely studied in the key proteins of the *γ*-secretase complex and even in the APP substrate, as well as their relationship with Alzheimer's disease, stability of complexes, and activity modulation [26, 27]. As presenilin proteins are catalytic subunits, they have diverse phosphorylation patterns and, according to the literature, they do not have a differential relationship mediated by cell localization, but there is an effect caused by their structure, despite the high homology between these proteins [26, 28–30]. The PS2 protein phosphorylations in the N-terminal fragment at positions Ser7, Ser9, and Ser19 are considered, from the protein kinases CK-1 and CK-2, whose function is to favor phosphorylation of a region close to the motif with a Pro-Glu-Ser-Thr (PEST) amino acid sequence that is related to protein turnover, but it is still unknown whether phosphorylation favors this turnover [28]. Other phosphorylation sites in PS2 consider the amino acids located in the caspase domains Ser327 and Ser330, which are involved in the inhibition of apoptotic properties of the caspase-mediated cleavages of PS2 [31]. The opposite is the case of the Asn141Ile mutation, whose phosphorylation is related to a modulating effect on the increase in the production of the 42 amino acid *β*-amyloid peptide [32, 33].

Computational approaches have been a great tool to find these effects on phosphorylations, for which there are various methodologies that provide algorithms derived from mega-alignments with PSI-BLAST, finding regions that are shared between proteins based on their primary sequence. Similarly, there are approaches through protein-protein interactions (PPI), associating groups in networks based on their function, and even three-dimensional regions relating protein folds [34–36]. In the study of macromolecular systems, mutations were analyzed from their functionality, taking into account simulations that consider changes at the topological and electronic structure level. Hybrid quantum mechanics/molecular mechanics (QM/MM) methods intended for biological systems at the macro scale have been widely used, as they allow experts to determine small structural changes due to nonsynonymous missense mutations, deletions, and insertions. There have also been studies considering an approach from a quantum mechanics (QM) perspective only, taking into account systems with more polarizable electrons such as methods of density-functional theory (DFT) with B3LYP bases or Hartree-Fock methods, which are very approximate methods for calculations regarding generation and breaking of bonds, such as the calculation of transition states and reaction coordinates [37–41]. They are, however, very expensive methods to study complex polyatomic systems and require very high computational resources; therefore, semiempirical methods are a very useful alternative, given that they allow for the study of systems in localized regions under a quantum mechanics description and the rest of the system is treated under a molecular mechanics description. Although from the quantum point of view there is not a very high level of theory, this is compensated with the possibility of analyzing macromolecular structures, taking into account the electronic correlation [42–44]. The semiempirical method has, among the best known bases, Austin model 1 (AM1), PM3, PM6, and MNDO, which are included in commercial software such as AMPAC, MOPAC, HyperChem, and Spartan [42, 45–48]. According to the literature, one of the most common bases in these systems, for energetic and geometric optimizations, is the AM1 basis, which is characterized by the fact that its parameters were derived from experimental data to simplify the approach to the Schrödinger equation [49–53]. As such, they can be applied with high efficiency to large molecules and in the calculation of the electronic structure of potential surfaces [42, 54–56].

In this work, we carried out a functional analysis of four mutations which are reported in the literature as having increasing effects on the amount of amyloid peptide, such as Met239Ile and Met239Val mutations located in the catalytic pocket. In the same way, Ser130Leu and Thr122 mutations were analyzed, in which there is not a very marked effect on canonical function, but an effect at the metabolic level with calcium concentration is reported. In view of the above, a simulation was made under a hybrid QM/MM methodology for the PS2 protein model proposed for the first time, studying effects at the topological level such as bond distances, angle changes, changes in electronic structure, and density surfaces with the aim of providing an explanation from a chemical visualization for the mutations that trigger Alzheimer's disease.

## 2. Methodology

### 2.1. Characterization Model in Databases

The PS2 protein was characterized from the information recorded in the databases, where it was found that its primary sequence has a size of 448 amino acids, its function is peptidase-like, it has three isoforms (the functional one being that containing 448 amino acids), and the amino acids of the active site (Aspartic acids) are in positions 263 and 366. In addition, at the experimental level, it does not have any crystal representing its structure, given that it has not been isolated by any experimental technique such as cryogenic imaging microscopy, X-ray diffraction, or solid-state nuclear magnetic resonance. The databases consulted were Uniprot, PSI (Protein model portal), EMBL, and Protein Data Bank without obtaining any structural information [57–61].

Upon conducting a literature review, four of the mutations reported therein for PS2 were selected, given the susceptibility they generate in the protein and the fact that they have already undergone experimental studies whose results show an increase in the production of amyloid plaques for mutations Met239Ile and Met239Val. The mutations showing alteration in calcium signaling were Thr122Arg, Ser130Leu, and Met239Ile [62].

### 2.2. Alignment of Primary Sequences

The PS1 and PS2 proteins are homologous in their protease function. Therefore, the alignment of the primary sequence for these two proteins is carried out in the FASTA format. With the Jalview software from the T-Coffee 2.0 and Clustal X tools, we obtain the quantitative values for the degrees of similarity between sequences, and this is congruent with the values provided by the literature. The alignment sequences are presented without a consensus histogram and taking into account the alignment quality histogram and the Logomat alignment of the sequence from its conservation [63, 64].

### 2.3. Structural Prediction Model

Structural prediction software has been used to build a hypothetical model of the PS2 protein, that is, there is no record of the structure of this protein in the Protein Data Bank (PDB-databank) [65]. As there is no crystallized structure, structural biology tools are used to generate the protein model, and two structure predictors, I-Tasser and Phyre2, are chosen for this. These software programs generate their models from an algorithm that allows for creating the hypothetical model, based on the homology comparison and folding recognition through the primary sequence, and these sequences are joined under a threading-type assembly [66–70]. The prediction models used, as a template, the subunit B of the CryoEM model for the PDB with ID 6IYC [71] and a complete artificial model of the PS1 protein that guides the generation of the missing flexible region. The MUSTER tool [72] performs the search and classifies these models within the template library to generate the modeling. Furthermore, the models undergo a structural refinement, based on subjecting the system to force fields, seeking to improve noncovalent interactions in the peptide backbone, explicitly hydrogen bonds in the assembly of the secondary structure and removal of steric hindrance, using REMO, Fragment-Guided Molecular Dynamics (FGMD), and ModRefiner software [73–76]. The loops created from the best validated model were refined with the Discrete Optimized Protein Energy method, which has a normalized value and belongs to the MODELLER software, under iterative cycles that consider spatial restrictions [77].

### 2.4. Viewer and Tridimensional Alignment

The structural visualization of the hypothetical models was carried out with the UCSF Chimera software, which has the capacity to recognize the files in PDB format provided by the different structural prediction software. The structural alignments are made from the Needleman–Wunsch global algorithm and the BLOSUM62 matrix [78], and from this 3D alignment it is intended to obtain the structural coincidence in most of the spatial distribution. Similarly, multi-alignment is used with the Visual Molecular Dynamics (VMD) software belonging to the NAMD-VMD package of the University of Illinois to measure the standard deviation of the model, the QH index (similarity coefficient and degree of similarity of the model) [79, 80].

### 2.5. Validation of the Prediction Model

The validation of the model was made using stereochemical and energetic tools, which aim to characterize the predicted hypothetical model. With regard to the stereochemical tools, the RAMPAGE software, which was developed at the University of Cambridge by the crystallography and bioinformatics group, is used for obtaining the different orientations and measuring the Phi (*φ*) and Psi (*ѱ*) angles of the dihedrals which constitute the planes involved in the protein peptide bonds [81]. The energetic validation is performed using the QMEAN_Disco_ score, which generates an estimate from the statistical potentials of the bonds belonging to the carbon skeleton that makes up the protein, and in the same way produces a normalized score value, where the values closest to one represent structures with more stable carbon skeletons [82, 83].

### 2.6. Bioinformatic Analysis of Variations in the Proteins

The selected mutations underwent a bioinformatic characterization whereby they were subjected to a phosphorylation prediction analysis (NetPhos 3.1) with 15 characteristic kinases of the soma. In addition, transmembrane passes were quantified from the hidden Markov models (TMHMM 2.0) and their location within the membrane [84].

### 2.7. Transmembrane Passes with the Hidden Markov Models

This software allows for an illustrative idea of the location of the PS2 protein in the lipid membranes. The calculation was made with TMHMM2.0 Suite Swiss-ExPASy, where the intra-extra cellular location of the amino acids is provided with a stochastic value for the formation of each transmembrane pass [84].

### 2.8. Prediction of Phosphorylation and Activation of Biochemical Pathways

This tool allows for calculating, by probability, the ease with which 15 of the most important kinases within humans can phosphorylate the evaluated protein. This phosphorylation allows comparison of the native protein with the mutated protein, generating a graph with the probability for each of the 15 kinases [85, 86]. The biochemical pathways are analyzed with the information recorded in the Reactome database for the pathway affected by the mutations evaluated [87–91].

### 2.9. Hydropathic Index of the PS2 Mutations Considered

The changes in polarity around the mutation location were calculated using Protscale (Swiss ExPASY suite), where the comparison between the native protein and the aberrant protein can be made, from the primary sequence in the FASTA format with the Kyte and Doolittle coefficients. Depending on the score obtained and the sign it has, a hydrophilic environment or a hydrophobic environment can be favored, with zero being the threshold that separates them [84].

### 2.10. Modeling of the Variants by the Hybrid Quantum Mechanics/Molecular Mechanics QM/MM Method

**Figure 1:**
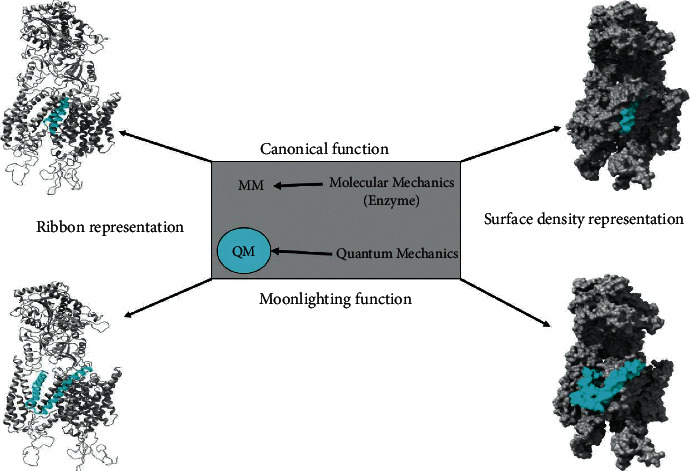
Cleavage regions considered with the hybrid QM-MM method for the PS2 protein in the *γ*-secretase enzyme: (upper) the region evaluated for canonical function; (lower) the region evaluated for the moonlighting function.

Macromolecular systems are analyzed from the application of various models of quantum theory to understand the effects of mutations or variants on the changes of the three-dimensional structure of proteins and the electronic structure. For this, the topological matrix is used. Furthermore, a semi-empirical method is used with the Austin model1 (AM1) basis of the Spartan'18 software from Wavefunction to measure energy changes, bond distances, and angle changes [45]. The QM fragment is the localized region where the mutation occurs (canonical or moonlighting), the MM region corresponds to the description of the rest of the enzyme under classical physics, and the QM/MM is considered the polarization of QM due to the classical MM region. In this method for geometric optimization using quantum mechanics, the optimization is made for the regions involved in the entry pore to the catalytic site. These regions are TM2 in positions 142–155, TM3 in the fragment 176–181, and TM5 positioned in 237–242, while 239 is the position where the mutation occurs. The simulation under the quantum approach uses a method with an AM1 theory level, as it includes a model with 1046 bases and 1144 electrons for its electronic description. Therefore, taking into account the computational resources at hand and the high calculation costs, high-level quantum theory methods cannot be easily applied. For the moonlighting subsystems, the TM1 and TM2 of PS2 are taken as study regions for the QM geometric optimization, which include positions 122 and 130. The total MM region of the system and the classical description of the QM region, for all the systems under analysis, is made with the MMFF_(aq)_ basis, estimating the second sphere of solvation under study. Moreover, the interface was saturated with hydrogen atoms, as presented in Figure 1 [92–98]. The total energy of the system is calculated under the subtractive formula of Maseras and Morokuma, as shown in equation (1):

(1) $$\begin{array}{c} E_{\mathrm{total}}=EQM+EMM_{\left(\mathrm{total}\right)}-EMM_{\left(\mathrm{QM}\right)}. \end{array}$$

In this equation, the total energy value of the models for the wild-type protein and the mutations is obtained under a multi-scale analysis in triplicate for each of the models, considering average energies to include them in equation (1) and their standard deviation for each of the determined systems [99, 100]. The surface simulation is done using the Surface tool, generating various surfaces such as density surfaces, potential-potential surfaces, ionization potential, and sweeps of electrostatic potential maps in ranges from −200 kJ/mol to 200 kJ/mol, with which the electronic distribution that favors phosphorylation-related anchoring is calculated [101].

## 3. Results and Discussion

### 3.1. Database Characterization of PS2

The existing literature on the PS2 protein suggests that it comes from a gene that is found on chromosome 1 with locus 1q31-1q42; when it is translated into a protein, it has a size of 448 amino acids in its wild structure. This protein, like PS1, is part of the *γ*-secretase complex and is the subunit that when assembled with three other protein subunits, such as niscastrin (NCT), anterior pharynx defective-1 (APH1), and presenilin enhancer (PEN-2), is in charge of the transmembrane protease cleavages of numerous substrates. Lastly, PS2 has its aspartyl-protease active site in the D263 and D366 aspartic acid amino acids; however, when searching for the crystallized structure of this protein, in databases such as UniProt, EMBL, Human Protein Reference, and Protein Data Bank, the results show that it has not been crystallized under any experimental technique to date. Considering this search in databases, the aim was to use structure predictors to generate a model proposal that, following an energetic and stereochemical validation, would be used in the functional study regarding AD and the relationship with its mutations.

### 3.2. Alignment with Software Jalview to Primary Sequences

**Figure 2:**
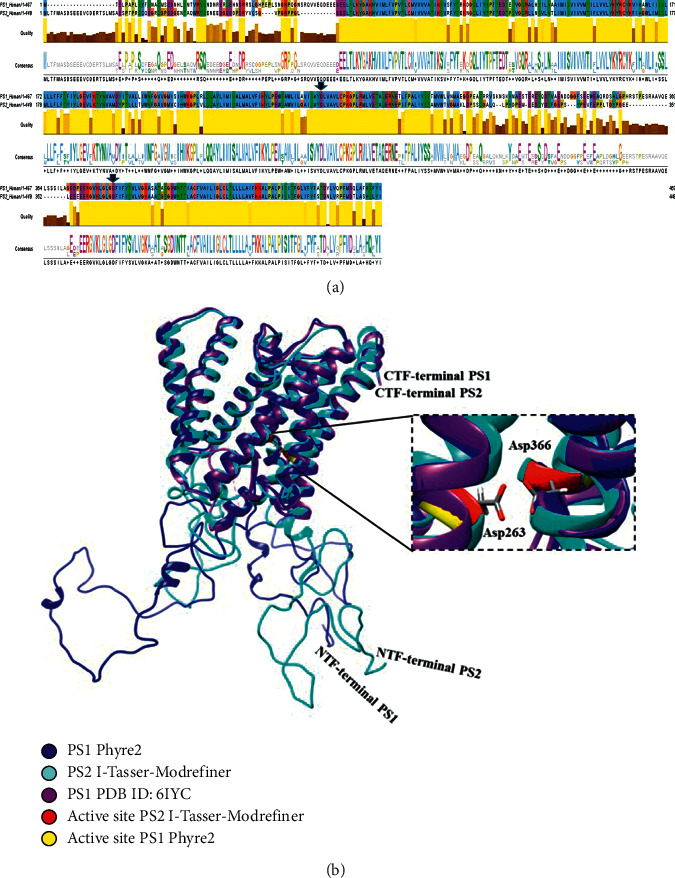
Alignment of the PS1 protein and the hypothetical model of PS2. (a) Alignment of primary sequences in FASTA. (b) Global three-dimensional alignment. Template protein PS1 Subunit B with ID: 6IYC (violet), complete model of protein PS1 with Phyre2 (dark blue), complete model of protein PS2 with I-Tasser and refinement FGMD (cyan), modeled active site of PS2 (red), and active site of PS1 (yellow).

The PS1 and PS2 proteins have a high degree of similarity in the peptidase active site, but from the FASTA sequence of the two proteins, the alignment of the primary amino acid sequences for the wild-type proteins is carried out through the T-Coffee 2.0 and Clustal X tools, where a value of ID 65.31 was obtained for the entire aligned sequence, as illustrated in Figure 2(a). In this image, the fragment of the active site of the protein is highly conserved (black arrow), since the two proteins coincide in aspartic acids D257 and D385 of PS1, and D263 and D366 of PS2. Moreover, when observing the histogram from the consensus, it is found that the largest “Logomat” alignment is in the structural fragment that shares the active site and that there is an important homology with an ID of 65.31. Furthermore, the quality of the alignment is shown as yellow histograms corresponding to the region of the protein with the highest homology and as brown histograms corresponding to the lowest alignment, according to the BLOSUM matrix 62. It is noted that the most variable fragments along with those which have the lowest quality response in the alignment by the amino acid pair are the Met1-Gly84 loop and the Gln282-Glu357 loop between the two *α*-helices that contain the active site. Consequently, we proceed to use two structure predictors with the respective refinement to evaluate and obtain a hypothetical model that describes in three dimensions what has been calculated with the primary sequences from the templates.

### 3.3. Construction of the Hypothetical Model from Structure Predictors

The topic of *in-silico* proposals for structure prediction based on the primary sequence has been widely explored in structural biology due to the identified need to create algorithms that allow for obtaining structural models of nucleic acids and proteins through comparison by homology, folding recognition, or de novo assembly. Proposing structural models for the functional study of some proteins lacking a resolved monocrystal structure is a good alternative. According to the structural models obtained, the effect of the refining in the noncovalent interactions that define the structure such as hydrogen bonds, hydrophobicity, hydrophilicity, or disulfide bonds is studied. Upon obtaining a score based on the quality of the model, both from the energetic and stereochemical points of view, the subsequent alignment is made with the reference obtained through cryogenic electron microscopy (CryoEM) at a resolution of 2.6 Å. This alignment is based on two protein structures; the first structure is a template reported in the PDB ID: 6IYC subunit B and the second structure is a homological model of PS1 protein, with the intention of comparing with the best predicted model of PS2 to verify the functional protease homology. In Figure 2(b), the three-dimensional alignment for the best selected PS2 prediction model is shown, with respect to the reference from the CryoEM structure of the PS1 released in the PDB with ID 6IYC and a complete hypothetical model of PS1 protein, taking into account the best values of the energetic and stereochemical validation for each model obtained. All these data were reported in Table 1.

According to the global alignment under the Needleman–Wunsch algorithm, it is possible to observe a very good spatial distribution of the topology of the prediction models with the CryoEM structure, showing that all the not-so-flexible regions have a high degree of similarity at the space level, while highly flexible regions, such as loops, differ in alignment values, and this fact is in line with the results of the alignment of the primary sequence. According to the comparison made with the T-coffee 2.0 tool, the same behavior is observed, where the best quality of the alignment and the lowest Logomat alignment are presented in these two regions of the FASTA comparison sequences for the two structures.

### 3.4. Multi-Alignment of Protein Structure and Sequences (STAMP)

Multi-alignment is a tool that allows for calculating the RMSD standard deviation of a model, the coefficient of similarity with the *Q*_*H*_ value, and the degree of similarity between the models. This alignment is carried out bearing in mind the various translations and rotations of the structures being compared. According to the results reported by each structure predictor software based on the modeling, we proceed to quantify the three-dimensional alignment of the best rated I-Tasser model with ModRefiner refinement, with respect to the 6IYC template and the complete PS1 artificial model template, as shown in Supplementary Figure 1(a). The alignment allows for a representation based on a color code for the structure, considering the respective standard deviation values and the structural fragment that is not easily aligned, while marking the areas of highest conservation. The structural representation model presents a standard deviation for the fragments of the model that are highly conserved and those that are not; these RMSD values are given according to the color code, as follows: high standard deviation and low conservation values (Red), standard deviation values between [2.0 and 2.5] Å (White), and values representing highly conserved fragments with low standard deviation (Blue). Using this procedure, the best alignment was obtained from comparing the 6IYC template of PS1 with the PS2 model of I-Tasser refined with ModRefiner with a quantitative *Q*_*H*_ value of 0.5043, RMSD 1.9672, and percentage identity of 48.48. In contrast, the template comparison of the complete artificial model of PS1 with the PS2 model of I-Tasser-Modrefiner presented a quantitative *Q*_*H*_ value of 0.3994, RMSD 2.3865, and percentage identity of 37.10.

With these results, the hypothetical model of PS2 with I-Tasser and ModRefiner refinement coincides better with the structure reported in the protein data banks since it presents a higher index of similarity in the three-dimensional alignment and a lower standard deviation between the models. The reported structure of PS1 was resolved by the cryogenic electron microscopy technique at a resolution of 2.6 Å for the region of the active site in the enzyme. The image Supplementary Figure 1(b) shows the comparison between the active site from the alignment of the primary sequence and the structural fragment of the active site shared by the proteins PS1 and PS2. Having selected the corresponding model and upon observing the coincidence in the active site, the idea of them being two proteins homologous in function that belong to different chromosomes is argued. Hereafter, we proceed to characterize the structural model to obtain acceptance based on the energetic and stereochemical validation; then, the results of the analysis obtained with the bioinformatics tools are presented.

### 3.5. Stereochemical and Energetic Validation of the Hypothetical Model of PS2

In order to be accepted, the structural model must meet certain validation parameters so that the hypothetical model can be used in the functional explanation of the nonsynonymous mutations of PS2 that, consequently, lead to the clinical manifestations of AD. The stereochemical validation is performed using Ramachandran plots, which allow for the quantification of the Phi (*φ*) and Psi (*ѱ*) angles that join the dihedral planes of the peptide bonds within the hypothetical structure. For the I-Tasser model refined using ModRefiner, the values obtained allow for locating the percentages of the various residues as follows: 86.3% in the favored region, 9.0% in the allowed region, 4.7% in the outlier region, while the values located in the regions of highest importance correspond to the residues that constitute the total of the PS2 protein of protease nature. The number of residues in the favored and allowed regions determines a good stereochemical validation and hosts a large proportion of residues in the quadrant, where there is an alpha-helix secondary structure.

The energetic validation is carried out with the QMEAN_Disco_ parameter, which uses statistical potentials from the single model method and restriction functions to calculate the scores corresponding to the carbon skeleton distances between the *α*-carbons, which constitute the protein throughout the peptide bonds. This value is normalized, and a value of 0.61 was established for the selected model.

### 3.6. Functional Analysis of Mutations in the Pore of PS2 Protein

The crystal of the PS2 protein has not been obtained at an experimental level, but from a previous modeling work, which used homology and structural prediction based on a template of the PS1 protein resolved by cryogenic electron microscopy. The characteristic of its homology in function reported in the literature is used to evaluate the functional effect of mutations reported in the literature at an experimental level. Regarding familial Alzheimer's disease, so far, PS2 has 52 reported mutations. A large proportion of these show incomplete penetrance, which means that, at a clinical level, people with the mutation will not necessarily develop the disease. Likewise, the *γ*-secretase enzyme not only processes the APP substrate but also another 93 different substrates. Thus, these small structural changes may be causing substrate interaction to be different between PS1 and PS2. The hypothetical model explains these protein-mediated functional changes in the enzymatic complex, at least from a theoretical standpoint [102–105].

The expression of PS1 (brain, gut, and vascular system) and PS2 (brain, liver, gut, and muscle) in diverse anatomical regions can explain why variations in *γ*-secretase substrates, such as Notch3, produce vascular effects by mutation in PS1 and not in PS2. Likewise, from a clinical standpoint, PS2 mutations have been related to cases of familial heart failure, which is correlated with the distribution and expression of one protein regarding the other. Moreover, according to the Human Protein Atlas project database and other repositories such as Tissue 2.0, higher levels of brain expression in PS1 compared to PS2 can explain why mutations in PS1 have greater pathogenic potential than those in PS2 [106–109].

PS2 mutations have been linked to greater breast cancer susceptibility through the Notch pathway, suggesting that the substrates cleaved by the *γ*-secretase enzyme may be altered. The Notch interaction is affected by these PS2 mutations, and considering that breast-cancer-related mutations are found in a site close to the protein's amino portion, these can probably generate an effect on the other subunits of the enzymatic complex (PEN-2, APH1, nicastrin), modifying the affinity for the substrate [103, 110, 111]. Furthermore, in clinical databases, PS2 mutations are considered to be a genetic risk factor for other diseases apart from Alzheimer's disease, such as Parkinson' disease, atypical dementia, frontotemporal dementia, among others [102, 112–114].

### 3.7. Structural Changes from the Hybrid Quantum Mechanics/Molecular Mechanics (QM-MM) Method

Quantum mechanics is a widely used tool in studies focused on protein systems. In the functional analysis of mutations reported in the pathology of AD, the hybrid QM/MM method can be coupled to the study of macromolecular complexes, and detailed follow-up can be carried out on the changes generated in the topological and electronic structure of various variants in PS2, such as Met239Ile, Met239Val, Ser130Leu, and Thr122Arg. The method used is semi-empirical, and the force field considers the AM1 basis to describe the electronic correlation in the system with parameterization based on experimental data.

### 3.8. Study of the Active Site of the PS2 Protein

**Figure 3:**
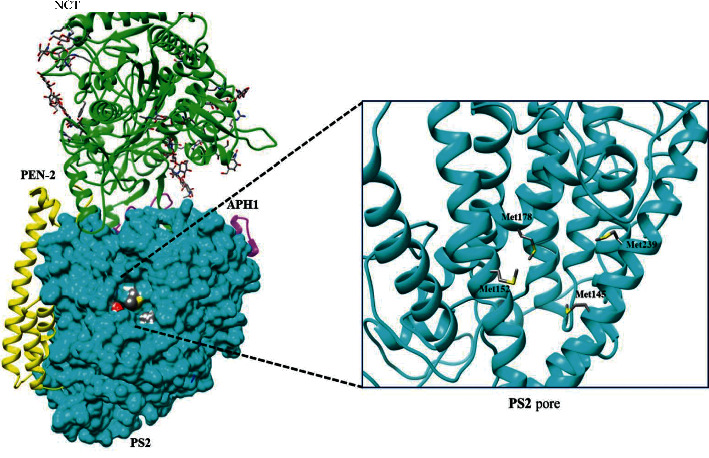
Visualization of the *γ*-secretase complex and ribbon representation of the first methionine access ring in Met145, Met152, Met178, and Met239.

With the aim of understanding the importance of position 239 in the protease function of the PS2 protein, it is noted that this position is part of a complex structure which has the methionine amino acids exposed to the outside of the protein pore. Although the chemical interaction between methionines and other amino acids is not known, the presence of this position could favor the regulation and entry of substrates so that the enzymatic complex can exert its protease function. The protein and the active site (Pore) are shown in Figure 3.

From the structural visualization, it is noted that before accessing the aspartyl-protease active site, there is a structure with methionines which, depending on their location, appears to be the first site of interaction with the substrates in the positions Met145, Met152, Met178, and Met239, where Met239, with two mutations, has a preponderant role in the diffusion of the substrate, given its possibility of altering the canonical function due to mutations in that location.

### 3.9. Topological Changes of Mutations Met239Ile and Met239Val in PS2

**Figure 4:**
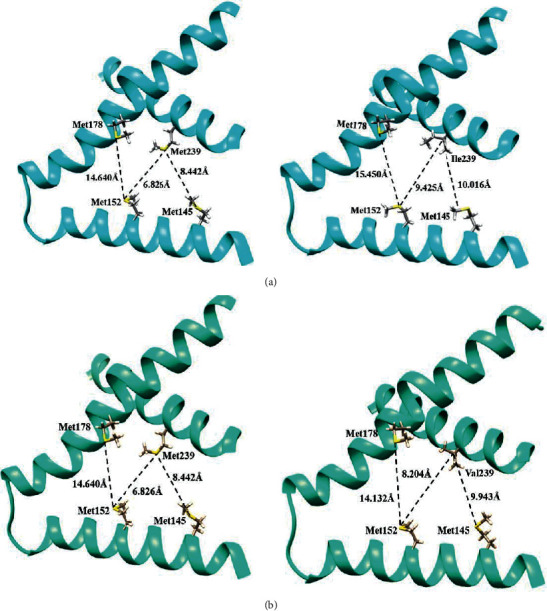
Structural changes in the pore of the PS2 protein by Met239Ile and Met239Val mutations. (a) Ribbon representation of the Met239Ile mutation. (b) Ribbon representation of the Met239Val mutation.

To understand the effect of mutations on the pore and a position exposed to the surface, structural characterization is carried out to observe the changes at the topological level of the mutations. The first mutation to be analyzed is Met239Ile. This mutation is characterized by an increase in hydrophobicity, which favors noncovalent interactions between nonpolar amino acids, such as London dispersion, and this interaction can generate a folding of the *α*-helix secondary structure. However, to quantify it, the wild-type and the variant proteins are modeled with the Spartan'18 software. With the aim of comparing the effects of the two systems, bond distances and density potential surfaces were measured to quantify the effect on the protein, surface areas, and surface volume.  Figure 4(a) shows the bond distances, considering the key positions of methionine for the mutation. The distances of the amino acids belonging to the wild-type PS2 protein were **Met178-Met152** 14,640 Å, **Met152-Met239** 6.826 Å, and **Met239-Met145** 8,442 Å. For the Met239Ile mutation, there was an increase in the pore distances with values of **Met178-Met152** 15.450Å, **Met152-Met239** 9.425 Å, and **Met239-Met145** 10,016 Å. The measured values show that when the mutation occurs, there is a large distance with respect to the wild-type protein, with values of 2 Å to 3 Å, favoring the diffusion of the substrate.

Met239Val mutation also occurs in an amino acid positioned in the pore and shows position 239, which is highly susceptible to aberrations. This change also generates an increase in hydrophobicity and plausible interaction with *α*-helices in the vicinities, which would produce a change in the pore size. Following energetic and geometric optimization, the calculation of the bond distances of the exposed amino acids in the pore is made. Also, the length values for the wild-type PS2 are obtained for the distances that conceive **Met178-Met152** 14.640 Å, **Met152-Met239** 6,826 Å, and **Met239-Met145** 8,442 Å. For the Met239Val mutation, the pore distances consider **Met178-Met152** 14,132 Å, **Met152-Met239** 8,204 Å, and **Met239-Met145** 9,943 Å. According to the results, it can be discerned that an increase in the distance from the entry region to the active site is occurring due to the structural change caused by the mutation. This is related to an increased production of amyloid peptide from a distance discrepancy in the reference points in the pore of approximately 1 Å to 2 Å, compared to the native structure of PS2, as shown in Figure 4(b).

### 3.10. Increased Production of Amyloid Peptide

**Figure 5:**
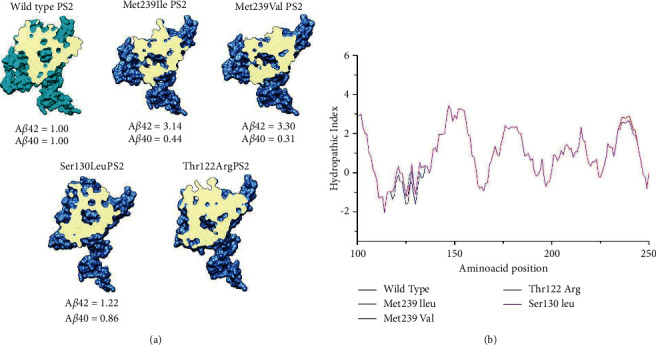
Analysis of selected mutations from various approaches. (a) Top view of the cross sections of the wild-type PS2 (pale blue), and the two mutations Met239Ile and Met239Val that affect the canonical function analyzed in the pore (dark blue). (b) Hydropathic index of the mutations evaluated in PS2.

This peptide is produced by the protease metabolism of the *γ*-secretase enzyme when, previously and extracellularly, the *β*-secretase enzyme cleaves the soluble fragment of APP, producing a carboxy-terminal fragment (CTF) of 99 amino acids. In the membrane, the *γ*-secretase performs the epsilon (*ε*) cleavage, and then successive cleavages until producing the gamma (*γ*) cleavage, where there is a variable proportion of *β*-amyloid fragments of sizes ranging from 43, 42, 40 to 38 amino acids. According to experimental and enzymatic studies, mutations in the homologous protein PS2 increase the amount of this peptide and are associated with Met239Ile and Met239Val mutations; hence, the effect they generate could be directly related to the diffusion of the substrate. When performing the energetic optimization of the native structure of PS2 and that of the mutated structure in the positions with the previous variant, there is an increase in the separation distance between a set of methionines that surround the active site of PS2. Moreover, it is possible to present cross-sections showing the increased size of the pore or pocket of the active site, as shown in Figure 5(a). From a chemical point of view, these mutations present very similar polarity changes as they both replace methionine, which has a thioether functional group by hydrocarbon chains in its side chain, and which does not have the possibility of generating any kind of electric dipole. The interaction forces that these nonpolar amino acids have are of the contact-area type, and hydrophobic interactions are favored, as shown by the calculation of the hydropathic index with the Kyte and Doolittle coefficients presented in Figure 5(b). The above prompts the mutation to interact much better with the constituent structure of the *α*-helices, and therefore, an opening is created in the channel, through which the processing of the substrate is carried out, which would increase its diffusion.

### 3.11. Electronic Changes of Mutations Ser130Leu and Thr122Arg in the PS2 Protein

**Figure 6:**
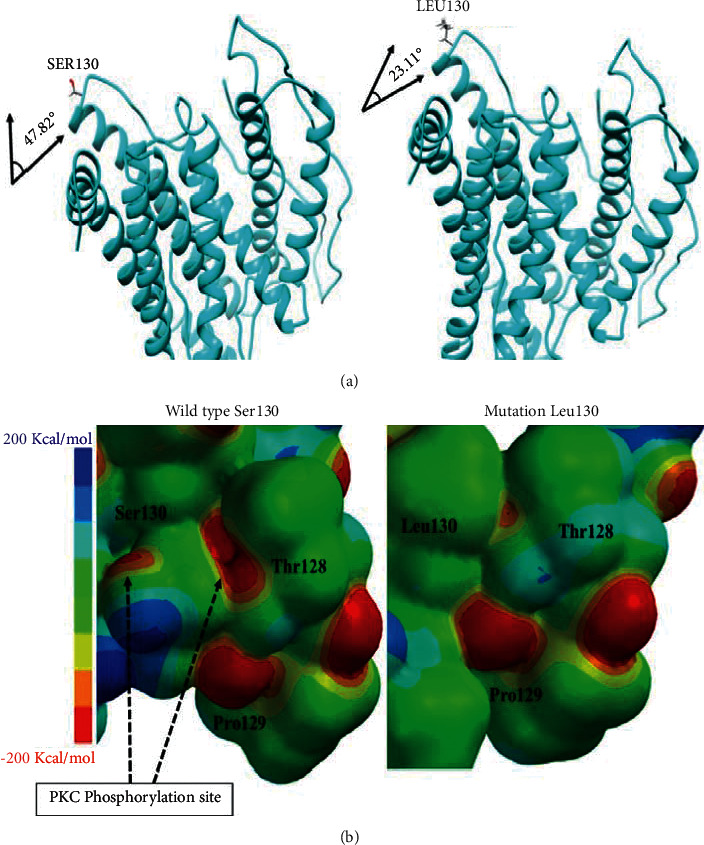
Structural and electronic changes of Ser130Leu mutation. (a) Topological changes of angle. (b) Electrostatic potential map in range of 200 kJ/mol to −200 kJ/mol.

In the case of the Ser130Leu variant, in which the effect of polarity change was clearly assessed, where a hydroxyl group is lost to obtain a noncovalent interaction by hydrogen bonding, the various hydrogen bonds were calculated under a restriction of 2.7 Å, using the UCSF Chimera software, where the binding lines that give orientation to the secondary structure are represented by red lines as shown in Supplementary Figure 2(a). From the results, we can observe changes in the hydrogen bond interaction patterns when the Ser130Leu mutation occurs in Supplementary Figure 2(b). This is because, quantitatively, there is a plausible point of interaction at a distance of 2.225 Å for the wild-type protein with the heteroatoms H-O to H-N, as shown in Supplementary Figure 2(a). When a change to nonpolar leucine occurs, the spot for hydrogen bonding is lost and the leucine side chain tends to seek hydrophobic interactions with other *α*-helices, favoring angle changes that bend the secondary structure, obtaining values of 47.82° for the wild-type N-C*α* Val131 compared to 23.11° for the N-C*α* Val131 mutation, for an angle difference of 24.71°, as shown in Figure 6(a).

These were the effects at the topological level, but changes in the electronic structure allow for the explanation of the metabolic imbalances caused by alterations in transduction signals due to the loss of phosphorylation. To do this, we first calculated the phosphorylation points of PS2 for the wild-type protein and the mutated Ser130Leu protein. The results showed that Ser130 is phosphorylated by the PKC kinase with a score of 0.627, whereas this phosphorylation is lost when the mutation occurs, and the PKC kinase communication is cut off. Similarly, with the semi-empirical method, the electrostatic potential map of both structures was calculated, determining the loss of one region of high electron density (represented in red) and obtaining a decrease in the surface electron density for the electrostatic potential with a value ranging from 50 to 100 Kcal/mol, losing the coupling site for the protein kinase, as shown in Figure 6(b).

**Figure 7:**
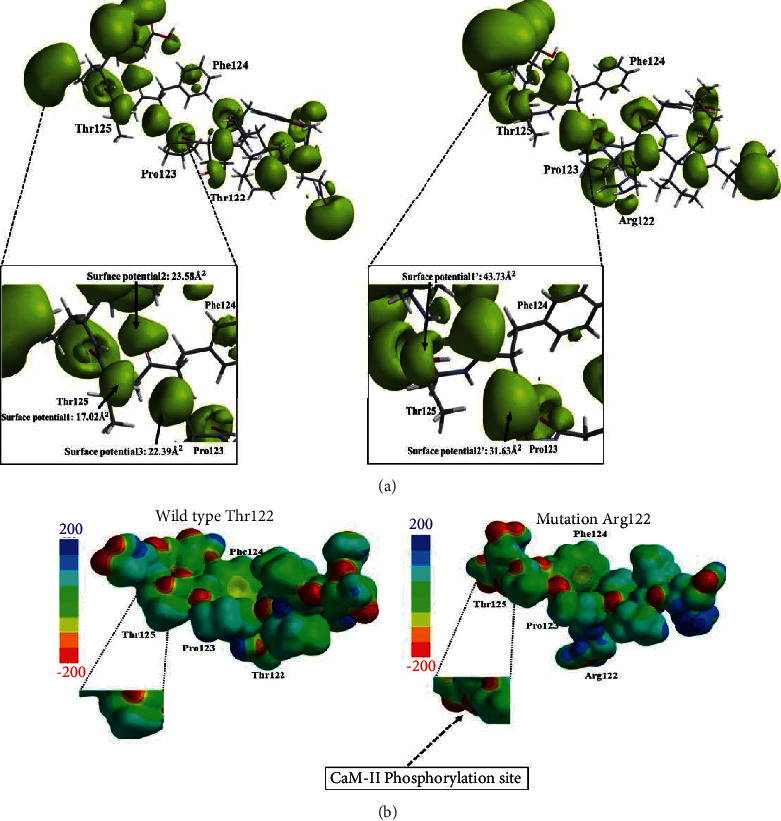
Structural and electronic changes of mutation Thr122Arg. (a) Potential-potential surfaces of wild type (left) and mutation Thr122Arg (right). (b) Electrostatic potential map for the vicinity of the position 122–126 in the guanidine-PS2 protein, wild-type protein (Left) and mutation Thr122Arg (Right).

The Thr122Arg mutation is a variant that produces an increase in polarity, given that it has a guanidine functional group which can generate an associated charge depending on the pH values of the medium. To understand the structural effect, a geometric optimization of the wild-type and mutated proteins was performed in the vicinity of position 122, also to understand the effect from the chemical visualization on the topology and electronic structure with the altered phosphorylation. When doing the energetic optimization of the molecular structure, the topological analysis of the wild type and the variant showed an evident change of angle for the two systems evaluated. This variant shows a difference of 15.23° between the 46.70° corresponding to the wild-type PS2 Thr122 protein and the 31.47° corresponding to the PS2 Thr122Arg protein, as represented with sticks in Supplementary Figure 3. This change has implications that not only affect spatial distribution but distort the electronic structure of the system. With the aim of explaining this, a potential-potential surface calculation was made, measuring the accessible area and specific area of the considered potential regions with respect to the variant position. The results of the calculation of density surfaces show that there is a notable change in the exposed area of the wild-type protein with respect to the mutated one.  Figure 7(a) shows the area calculated for each region in the vicinity of position Thr122, where the following potential points are considered: surface potential1: 17.02 Å2, surface potential2: 23.5802 Å2, and surface potential3: 22.3902 Å2. Later, the same study was done regarding the vicinity of the Arg122 mutation, determining that there is a unification of two density surfaces, which are considered as surface potential1': 43.7302 Å2, and surface potential2': 31.63 Å2. Compared to the wild-type PS2, the Thr122Arg mutation presents an increase of 3.13 Å^2^ in the relative potential surface. The difference obtained from unifying the two potential areas in the mutation represents an increase with respect to the wild-type protein. Moreover, the lower potential surface near the access point in Pro123 also increases with a difference of 9.24 Å^2^. Furthermore, calculations showed that the surface area of the entire wild-type potential-potential surface had a value of 630.05 Å^2^, the area accessible by another potential interaction surface had a value of 74.09 Å^2^, and the volume of the potential surface for the PS2 protein had a value of 282.03 Å^3^. Consequently, regarding the PS2 Thr122Arg mutation, the surface area of the mutated potential-potential surface was 669.02 Å2, the area accessible by another surface was 77.69 Å^2^, and the volume was 304.60 Å3, which favors phosphorylation in the mutation. This means that the area surrounding the mutation in position 122 gains potential and can easily distribute the electrons of the Thr125 hydroxyl group in the “d” orbitals of phosphorus and activate an alternate pathway of cellular signal transduction, allowing phosphorylation. With these results, we carried out the respective discussion on each of the mutations involved and their approach to molecular visualization, understanding the effect on the electronic structure of the PS2 protein.

### 3.12. Protein Moonlighting and Phosphorylation Effect on the Alteration of Signal Transduction

When analyzing the results obtained, and understanding the implication in calcium metabolism that, according to the literature, is exerted by mutations Thr122Arg and Ser130Leu, it is not possible to analyze the protease function. This is not possible because, according to the spatial distribution and its topology, there is no relation between its location and the mobility within the pore and the cleavage of the substrate. Therefore, given that some genes present pleiotropy and, following transcriptional maturation and subsequent translation, manifest more than one function in the proteins, we proceeded to analyze the effect of this protein and its phosphorylation on signal transduction at the cellular level to observe if there is a marked effect in this respect.

### 3.13. Phosphorylation Effect of Mutation Thr122Arg

In understanding the structural effect of the PS2 protein mutation, it is noted that, from its position at 122, there is no remarkable change in the enzymatic activity of the *γ*-secretase complex, as it is far from the active site. Hence, the focus of study is oriented towards understanding the effect of phosphorylation on the metabolic activity since it is possible to observe the phosphorylation blockage or nonblockage in the electronic structure. This variant is considered a change in which there is an increased polarity, as shown in Figure 5(b), which means that the surroundings of the mutation increase its polarity and may expose more kinase anchoring sites with charged environments or sites for dipole-dipole type interaction forces.

Threonine is an amino acid that has a hydroxyl group in its side chain; this functional group has pairs of free electrons which can be used within the nucleophilicity of the system to phosphorylate and attack the phosphorus electrophile, taking advantage of its oxophilicity to be bound and be mediated by protein kinases. When a mutation is generated at that point, it is important to bear in mind that phosphorylation may be lost, which has been corroborated through bioinformatic calculations from the NetPhos 3.1 Server software. There is a change which involves going from having a cdk5 kinase phosphorylation with a score of 0.513 for position Thr122 to not having any kinase phosphorylation at that point. Interestingly, it has been calculated that despite the loss of kinase phosphorylation at Thr122, when the Arg122 mutation is generated, an angle change which could be measured by energetic optimization, based on the hybrid Quantum Mechanics-Molecular Mechanics method, allowed for establishing that phosphorylation was favored at position Thr125, which did not occur in the wild type. This was better clarified upon taking into account the calculation of surfaces for the vicinity of position 122, as well as position 125, since the idea is to illustrate the effect on the mutation surface and its plausible phosphorylation blockage. As a remarkable result, it was possible to confirm that from the selected measurement of the accessible area and specific area, an increase is generated around position 125, which can be complemented using bioinformatics tools. When measuring the phosphorylation of the mutation with the ExPASy software, it was determined that this modification made the protein lose a phosphorylation site. However, it gained a site for Thr125 with a score of 0.712, for an unspecified kinase that can be configured for the protein calmodulin II (CaM-II), which then appears in second place with a score of 0.455 for CaM-II. This fact generates an alteration in cell signaling, due to a signal transduction resulting from the phosphorylation in another position, which did not occur in the wild-type protein. This kinase has an important role in calcium metabolism, given that CaMKII is involved in several cell signaling cascades and is an important mediator in learning and memory processes. Multifunctional CaM-II kinases also play an important role in numerous processes, such as neurotransmitter release, transcription factor regulation, and glycogen metabolism, as shown in Figure 7(b).

### 3.14. Phosphorylation Effect of Mutation Ser130Leu

When studying the mutation and its relationship with the clinical manifestations of the disease, where the variant facilitates the development of Alzheimer's disease, the protease function is again not greatly affected. Therefore, the analysis is made based on the structure of the serine residue in the wild-type protein, which has a hydroxyl functional group in its side chain that is considered a primary alcohol instead of a secondary one as in threonine and whose characteristic allows it to have its pair of electrons more available to add them in a nucleophilic way to the electron-deficient zone of phosphorus, generating a phosphorylated bond mediated by protein kinase. According to the bioinformatic calculation, serine is phosphorylated at 130 in the wild-type protein, but this phosphorylation is lost when the mutation occurs at 130 to Leu, which is a nonpolar amino acid that shows, for instance, the loss of a key anchoring point and hydrogen bond interaction with glycine at position 132.

When calculating phosphorylation for the Ser130 wild-type position through bioinformatic tools intended for the Swiss ExPASY suite software, it was found that its phosphorylation score was 0.627 for two PKC kinases and 0.649 for one Unspecified kinase, whose values are lost when the mutation to Leu130 occurs at that position. Similarly, it is observed that this variant does not affect the position close to phosphorylation in Thr128, where, in the case of the wild-type and the mutated proteins, the unspecified kinases with a score of 0.960, and the p38MAPK with a score of 0.501, do not change.

To validate the data generated from phosphorylations, we proceeded to study, by means of surface calculation, whether there is a structural effect that shows the loss of phosphorylation. Therefore, upon doing the energetic optimization, according to the electrostatic potential map, it is noted that there is a loss of regions of high electron density distributed which have an electrostatic potential value that moves from −200 kJ/mol to areas that range from −50 kJ/mol to 50 kJ/mol. The visual effect contemplates that the loss of regions of high electron density disfavors the possible anchoring points for PKC and unspecified kinases. In addition, when evaluating the conservation effect in the points surrounding position 130, the red points in the zones of high electron density at position Thr128 are not lost. Hence, there likely exists an effect on metabolism that may be related to low calcium homeostasis due to the loss of phosphorylation at 130.

### 3.15. Energetic Calculations of the Canonical and Moonlighting Function Systems

Based on the systems studied, the approach referring to the canonical function, as well as the changes in amyloid peptide production and in calcium metabolism mediated by cell signaling, we carried out the quantitative calculation of the system's energies, for specific regions, in the electronic structure changes corresponding to the canonical and moonlighting functions. The energies for each of those regions are shown, in kJ/mol, in Table 2, where the data of the energetic and geometric optimizations, as well as the position of the study, are recorded.

The above table shows the values reported, in triplicate, for the three subsystems evaluated, analyzing the topological and electronic structure as tools to understand protein functionality in the PS2 model. The values of the simulation show that, in the canonical function for mutations that are just at the entrance of the pore, there is a slight change in the energy values, which results in an increased size for the catalytic pocket of the PS2 protein. Similarly, both mutations in 239 are associated with a change in polarity which can be related to changes in the structure of the pocket, which favor the cleavage by means of the pathway that produces the most hydrophobic peptide of A*β*42 in a higher proportion, as observed in Supplementary Table 1(a). When calculating the delta values for each of the most frequent peptide fragments, we found that for the A*β*42 peptide there is an increase of 2.14 in the M239I mutation, more than twofold when compared to the wild type, and a decrease of −0.56 for the A*β*40 fragment. Furthermore, the same happens with the M239V mutation, where there is an increase of 2.30 in the A*β*42 fragment compared to the wild type; and a decrease of −0.69 for the A*β*40 fragment. Taking these data into account, changes in polarity favor the cleavage pathway that leads to the production of the 42-amino acid fragment over that of the peptide of 42 amino acids. These changes also increase its production, which can trigger a greater possibility of oligomerization of fibrils and generation of senile plaques.

**Figure 8:**
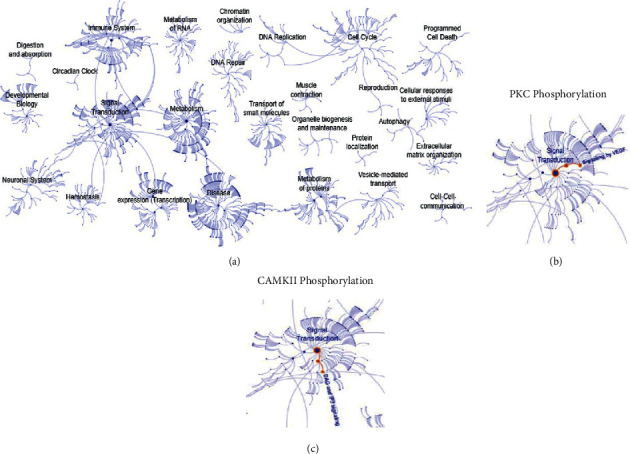
Characterization of metabolic pathways with the Reactome tool. (a) Metabolic pathways stored for *Homo sapiens*. (b) Transduction pathway turned on by PKC phosphorylation. (c) Transduction pathway by CAMKII phosphorylation.

Regarding position 130, the total energy change is not so drastic in the simulation; even though it did not show very significant changes in the total production of the amyloid peptide, the increased hydrophobicity made the amount of A*β*42 vary again, thus this was greater than the amount of A*β*40 peptide. To understand this, in Supplementary Table 1(b), the delta of change is also calculated for the S130L mutation, which generates a value of 0.22 for the A*β*42 fragment and a value of −0.14 for the A*β*40 fragment. This allows for understanding that the canonical function is not very affected by this mutation, and the pathogenesis of the disease must be due to other mechanisms. In this scientific paper, the group of associate researchers proposes a plausible explanation at the theoretical level by analyzing cell signaling, considering the signal transduction from the change in the respective phosphorylation patterns, and monitoring whether the response signal is lost or new patterns appear. The study determined that, for this specific S130L mutation, there is a signal loss for the phosphorylation via PKC kinase when compared to that of the wild type. The above takes into account the calculation of signaling pathways in *Homo sapiens* made in the Reactome database, as shown in Figure 8(a), emphasizing the activation of the VEGF signaling pathway due to phosphorylation at Ser130 for the signal transduction subset, as presented in Figure 8(b).

In the case of the T122R mutation, there are no reported data regarding the amount of amyloid produced, as presented in Supplementary Table 1(c). Hence, the delta could not be calculated and compared with the other mutations, but according to the clinical picture reported in the literature, this mutation is associated with changes in calcium metabolism. When measuring the hydropathic index of the wild-type and mutated PS2 proteins using the Kytte and Dolittle coefficients, it is possible to observe that there is an increased hydrophilicity associated with the mutation, which can trigger changes at the structural level marked by delta values close to those of the wild type. From the computational simulation, it is possible to note a slight decrease in total energy; therefore, an analysis was carried out from the phosphorylation patterns. Such analysis found that for the wild-type PS2 there was a phosphorylation point at the Thr122 position, which is lost when the mutation occurs. However, for positions in the vicinity of 122, the change in polarity leaves position 125 much more exposed; this position is at the extracellular level and is favored to undergo phosphorylation, which does not happen for the wild type. The pathway that is activated is the CAMKII-dependent pathway, which is involved in calcium metabolism and is responsible for signal transduction for the DAG and IP3 pathways, as shown in Figure 8(c). This proposal, which was analyzed from the post-translational modifications of the proteins, was supported by the surface analysis carried out. With the metabolic pathways activated, according to the databases stored in Reactome, the signal transduction processes may be dedicated to transmitting effector signals intended to elicit a response at the cellular level or not.

According to the images obtained, the characteristic transduction pathways are those that can be mediated by PKC phosphorylations, which activate the VEGF (vascular endothelial growth factor) metabolic pathway, responsible for cell migration and vascular cytoprotection in the face of ischemia, reperfusion, endothelialization, and shock. The pathway that activates CAMKII transduces the signal of the DAG and IP3 pathways, which are responsible for promoting the movement of calcium into the cytosol, thus regulating its intracellular concentration, and have stimulating functions in lipid metabolism, proliferation, cell differentiation, programmed cell death (apoptosis), and neurotransmission.

## 4. Conclusion

Many functional studies on protein systems have many limitations at the experimental level, given that they depend on the existence of the crystal and its topology. Molecular models built with structure predictors are an alternative approach to the study of variants that affect the chemical environment of the system under study.

The structure of the presenilin-2 model is solved from homology comparisons with the primary sequences, folding recognition, and generation of new structureless fragments from *ab-initio*, subjecting the amino acids in their peptide bonds to force fields.

The study of the structural relationship and its various effects on the clinical picture of AD includes mutations that increase production of the amyloid peptide, thus increasing the number of oligomers in peptides of various sizes and favoring the generation of senile plaques. Likewise, the imbalance of calcium metabolism in the neuronal cell is considered. This information was reported in the literature by experimental studies that did not have an illustrative model of the system. When trying to find an explanatory model of these variants, it was calculated that there is a differentiation in the effects of the mutations considered, such as Met239Ile and Met239Val. An increased distance in the pore entry was also found for the APP substrate with respect to the wild type, suggesting that there is a greater diffusion within the active site of the PS2 protein that has its aspartyl-protease active site in D263 and D366. A study is also carried out to understand the impact on calcium metabolism associated with mutations Ser130Leu and Thr122Arg, where, from its location, a direct impact on the protease function is not conceived. Then, the effect on signal transduction is analyzed, and the results showed that phosphorylation blockages occur because of the loss of regions of high electron density, which are crucial for enzymatic anchoring with kinases. As with the surface analysis, it was also found that the activation of other phosphorylation sites can be obtained in substitution to the lost sites, and this can be quantified through changes in the accessible area, surface area, specific area corresponding to the position of surface potentials, and the volume variations. The assembly of the enzymatic complex is important for enzyme activity, and studying the interactions or effects of the mutations on the vicinities of the NCT, APH1, and PEN-2 *γ*-secretase subunits remains as a future prospect. There are very few studies on this subject, and it is essential to characterize the other regions of the protein distant from the active site.

## Figures and Tables

**Table 1:** Energetic and stereochemical validation of the created models of the PS2 protein.

Model	Software	QMEAN_Disco_	Ramachandran plot
PS2 unrefined	I-tasser	0.539	Favored region: 381 (85.4%)
			Allowed region: 52 (11.7%)
			Outlier region: 13 (2.9%)

PS2 refined	I-tasser-ModRefiner	0,598	Favored region: 419 (93.9%)
			Allowed region: 19 (4.3%)
			Outlier region: 8 (1.8%)

PS2 refined	I-tasser-remo	0.536	Favored region: 381 (85.4%)
			Allowed region: 52 (11.7%)
			Outlier region: 13 (2.9%)

PS2 refined	I-tasser-FGMD	0.550	Favored region: 385 (86.3%)
			Allowed region: 40 (9.0%)
			Outlier region: 21 (4.7%)

PS2 unrefined	Phyre2	0.511	Favored region: 386 (86.5%)
			Allowed region: 38 (8.5%)
			Outlier region: 22 (4.9%)

PS2 refined	Phyre2-ModRefiner	0.528	Favored region: 401 (89.9%)
			Allowed region: 32 (7.2%)
			Outlier region: 13 (2.9%)

PS2 refined	Phyre2-remo	0.388	Favored region: 345 (77.4%)
			Allowed region: 39 (8.7%)
			Outlier region: 62 (13.9%)

PS2 refined	Phyre2-FGMD	0.451	Favored region: 350 (78.5%)
			Allowed region: 66 (14.8%)
			Outlier region: 30 (6.7%)

**Table 2:** Energy values calculated in the system with PS1 in the *γ*-secretase enzyme.

**Protein PS2**	**Energy MM (full length)**	**Average MM (full length)**	**Energy MM (QM)**	**Average MM (QM)**	**Energy QM**	**Average QM**	**Total energy: EQM** **+** **EMM-EMM(QM)**
**Canonical function of protein TM2-tm3-tm5**							
**Wild type1**	336896.0128	336896,0120 (±) 0,0020	865.2985	865,2980 (±) 0,0040	−7488.8253	−7496,0 (±) 9,0	328535,0 (±) 9,0
	336896.0131		865.3022		−7493.211		
	336896.0100		865.2945		−7506.6598		
**Met239Ile**	339314.9126	339314,910 (±) 0,020	818.4832	818,4834 (±) 0,0040	−6870.5981	−6867,0 (±) 11,0	331630,0 (±) 11,0
	339314.8922		818.4870		−6853.8652		
	339314.9296		818.4801		−6875.5497		
**Met239Val**	339272.8582	339272,8580 (±) 0,0020	895.2571	895,2570 (±) 0,0020	−7613.9552	−7602,0 (±) 18,0	330776,0 (±) 18,0
	339272.8561		895.2548		−7580.6855		
	339272.8599		895.2591		−7609.8468		

**Moonlighting function of protein TM1-TM2**							
**Wild type2**	336896.0128	336896,0129 (±) 0,0080	−137.9956	−138,000 (±) 0,020	−2408.1199	-2408,1150 (±) 0,010	334625,8930 (±) 0,0050
	336896.0051		−137.9817		−2408.1026		
	336896.0209		−138.0089		−2408.1224		
**Ser130Leu**	337994.3584	337994,40 (±) 0,10	−181.1549	−181,1550 (±) 0,0070	−2277.7856	−2277,7820 (±) 0,0030	335897,70 (±) 0,10
	337994.4981		−181.1619		−2277.7798		
	337994.2197		−181.1481		−2277.7798		

**Moonlighting function of protein TM1-TM2**							
**Wild type3**	336896.0128	336896,0120(±) 0,0020	−60.0502	−60,0503(±) 0,0020	−3585.7869	−3585,90 (±) 0,10	333370,20 (±) 0,10
	336896.0131		−60.0488		−3586.0359		
	336896.0100		−60.0519		−3585.8762		
**Thr122Arg**	321022.4972	321022,50(±) 0,50	−812.7245	−812,7240(±) 0,0040	−3264.0175	−3256,0 (±) 7,0	318580,0 (±) 8,0
	321022.9894		−812.7284		−3250.9481		
	321022.0045		−812.7202		−3251.5557		

## Data Availability

The datasets generated and analyzed during the current study are available from the corresponding author on reasonable request.

## Abbreviations

AD: Alzheimer's disease
PS2: Presenilin-2
PS1: Presenilin-1
APP: Amyloid protein precursor
MAPT: Microtubule-associated protein tau
LOAD: Late onset Alzheimer's disease
EOAD: Early onset Alzheimer's disease
NCT: Nicastrin
APH1: Pharynx-defective-1
PEN-2: Presenilin enhancer-2
STAMP: Multialignment of protein structure and sequences
RMSD: Root mean square deviation
FGMD: Fragment-guided molecular dynamics
SNARE: Soluble attachment protein receptor
PKC: Phosphokinase C
QM: Quantum mechanics
MM: Molecular mechanics
cdk5: Cell division protein kinase 5
uniprot: Universal protein
EMBL: European molecular biology laboratory
PSI: Protein model portal
Blosum: Blocks of amino acid substitution matrix
TMHMM: Transmembrane hidden Markov model
TM: Transmembrane
AM1: Austin model1
CryoEM: Cryogenic electron microscopy.

## Supplementary Materials

Supplementary Materials Supplementary Figure 1: alignment of PS1 protein with PS2: (a) quantitative multi-alignment of proteins at 3D level using color coding: values are considered in the alignment (red) with high standard deviation (RMSD), standard deviation in the range of [2.0–2.5] Å (white), and highly conserved and low standard deviation regions (blue); (b) active site alignment of the primary amino acid sequence of PS1 with PS2. Supplementary Figure 2: topological and electronic structure change of the Ser130Leu mutation: (a) hydrogen bonds calculation (red) in ribbon representation of the wild-type PS2 protein; (b) hydrogen bonds calculation (red) in ribbon representation of the Ser130Leu PS2 protein mutation. Supplementary Figure 3: angle change for PS2 wild-type protein (left) and Thr122Arg mutation (right). Supplementary Table 1: energetic values calculated in the system with PS2 in the *γ*-secretase enzyme: (a) energy average of canonical PS2 function and M239I, M239V mutations; (b) energy average and characterization for the wild-type and mutated moonlighting function Ser130Leu; (c) energy average and characterization for the moonlighting wild-type and mutated Thr122Arg function.
