# Supplementary material for: Structural Predictive Model of Presenilin-2 Protein and Analysis of Structural Effects of Familial Alzheimer's Disease Mutations
Source: Biochem Res Int. 2021 Nov 29;2021:9542038. doi: 10.1155/2021/9542038 (PMC8648483; doi:10.1155/2021/9542038)
Supplement: Supplementary Materials — Supplementary Figure 1: alignment of PS1 protein with PS2: (a) quantitative multi-alignment of proteins at 3D level using color coding: values are considered in the alignment (red) with high standard deviation (RMSD), standard deviation in the range of [2.0–2.5] Å (white), and highly conserved and low standard deviation regions (blue); (b) active site alignment of the primary amino acid sequence of PS1 with PS2. Supplementary Figure 2: topological and electronic structure change of the Ser130Leu mutation: (a) hydrogen bonds calculation (red) in ribbon representation of the wild-type PS2 protein; (b) hydrogen bonds calculation (red) in ribbon representation of the Ser130Leu PS2 protein mutation. Supplementary Figure 3: angle change for PS2 wild-type protein (left) and Thr122Arg mutation (right). Supplementary Table 1: energetic values calculated in the system with PS2 in the γ-secretase enzyme: (a) energy average of canonical PS2 function and M239I, M239V mutations; (b) energy average and characterization for the wild-type and mutated moonlighting function Ser130Leu; (c) energy average and characterization for the moonlighting wild-type and mutated Thr122Arg function. [file 9542038.f1.pdf]

Hindawi

Supplementary Material

Structural predictive model of Presenilin-2 protein and analysis of structural effects of familial Alzheimer's disease mutations

Alejandro Soto-Ospina[1,3], Pedronel Araque Marín[2*], Gabriel de Jesús Bedoya[1] and Andrés Villegas Lanau[1,3*]

[1]University of Antioquia, Faculty of Medicine, Group Molecular Genetics, Medellín, Colombia.

[2]EIA University, School of Life Sciences, Research and Innovation in Chemistry Formulations Group, Envigado, Colombia

[3]University of Antioquia, Faculty of Medicine, Group Neuroscience of Antioquia, Medellín, Colombia.

***To whom correspondence should be addressed:**

Alejandro Soto Ospina, e-mail: johnny.soto@udea.edu.co

University of Antioquia, Faculty of Medicine, Group Molecular Genetic (GenMol), Calle 62 #52-59 Laboratory 430, Medellín - Antioquia, Colombia.

Andrés Villegas Lanau, e-mail: andres.villegas@udea.edu.co

University of Antioquia, Faculty of Medicine, Group Molecular Genetic (GenMol), Calle 62 #52-59 Laboratory 411-412, Medellín - Antioquia, Colombia.

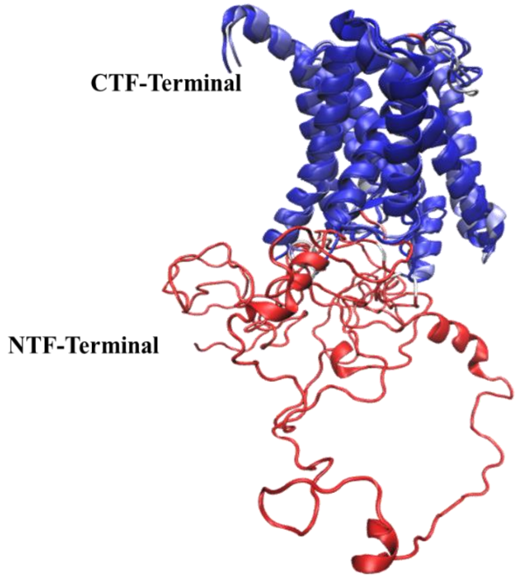

A

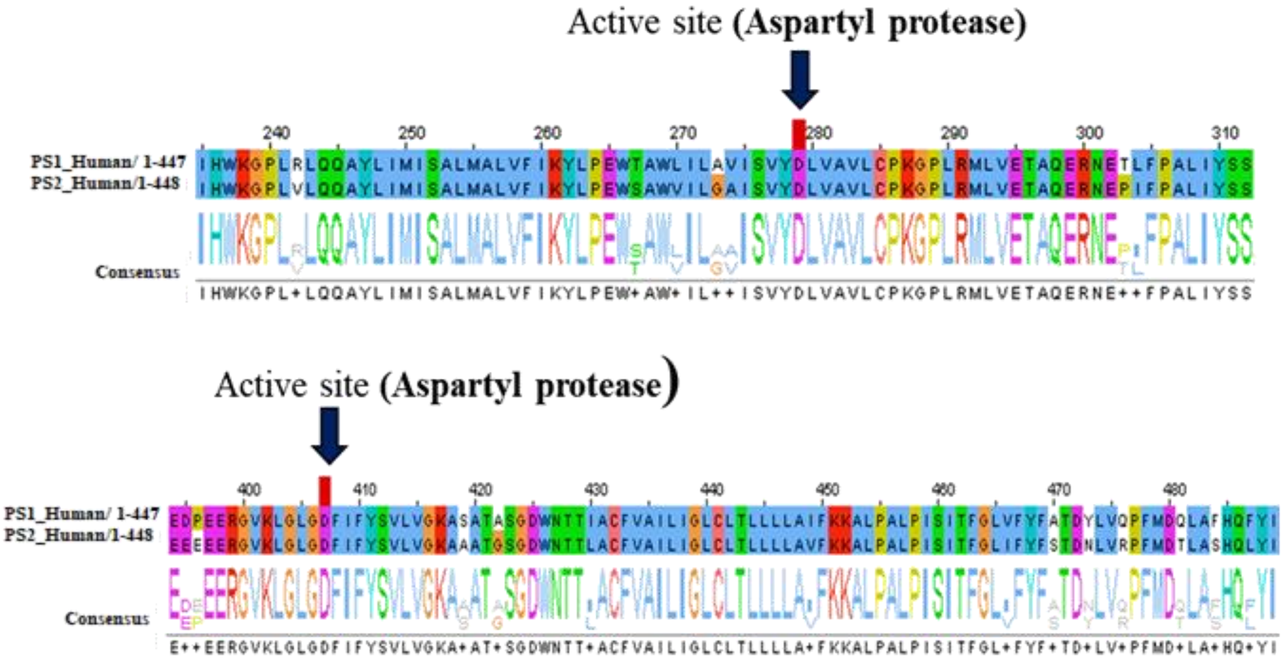

B

Sup.Figure 1. *Alignment of PS1 protein with PS2: A) Quantitative multi-alignment of proteins at 3D level using color coding: values are considered in the alignment in (Red) with high standard deviation (RMSD), standard deviation in the range of [2.0-2.5] Å (White), highly conserved and low standard deviation regions (Blue); B) Active site alignment of the primary amino acid sequence of PS1 with PS2.*

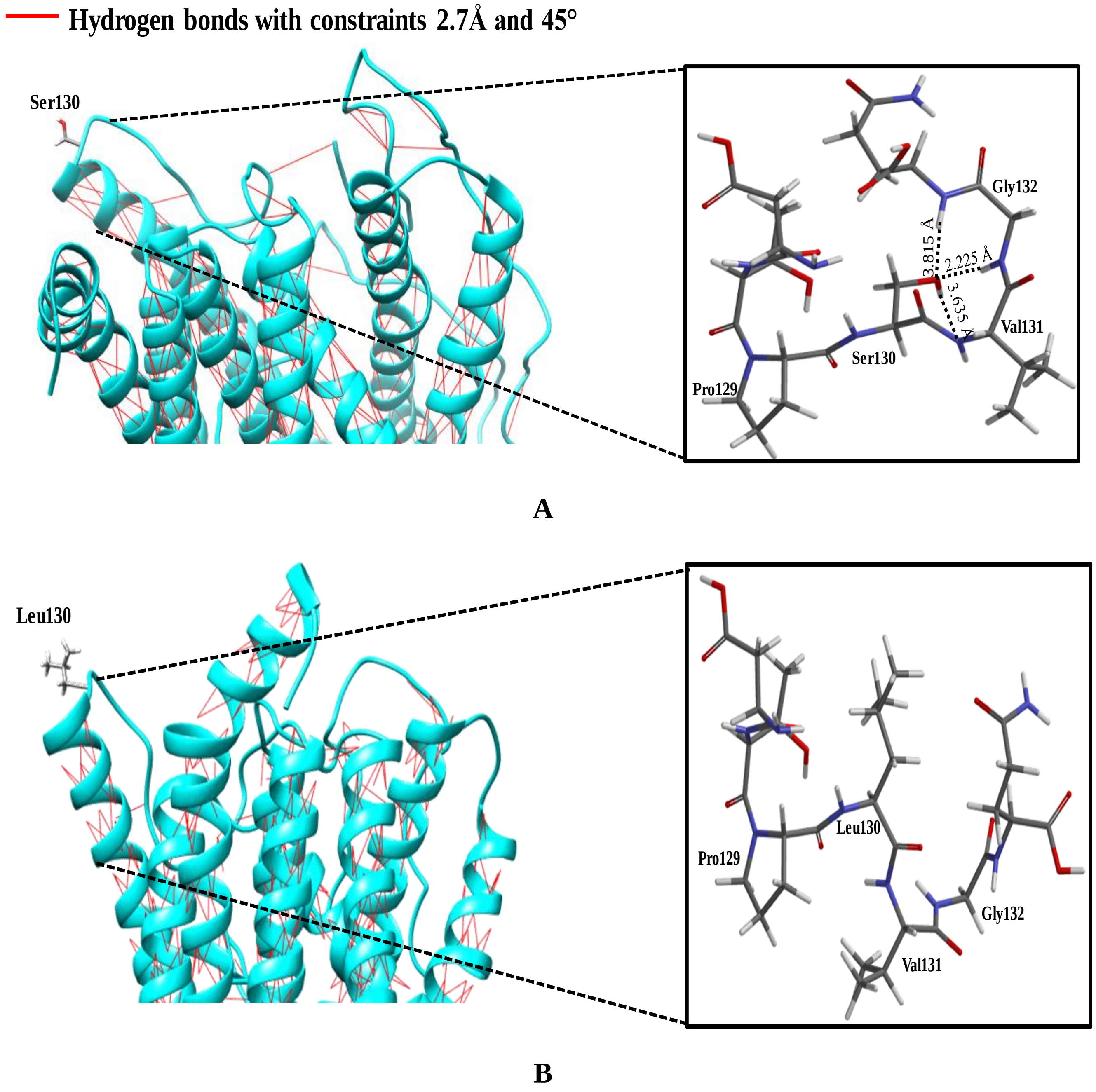

Sup.Figure 2. *Topological and electronic structure change of the Ser130Leu mutation: A) Hydrogen bonds calculation (red) in ribbon representation of the wild-type PS2 protein; B) Hydrogen bonds calculation (red) in ribbon representation of the Ser130Leu PS2 protein mutation.*

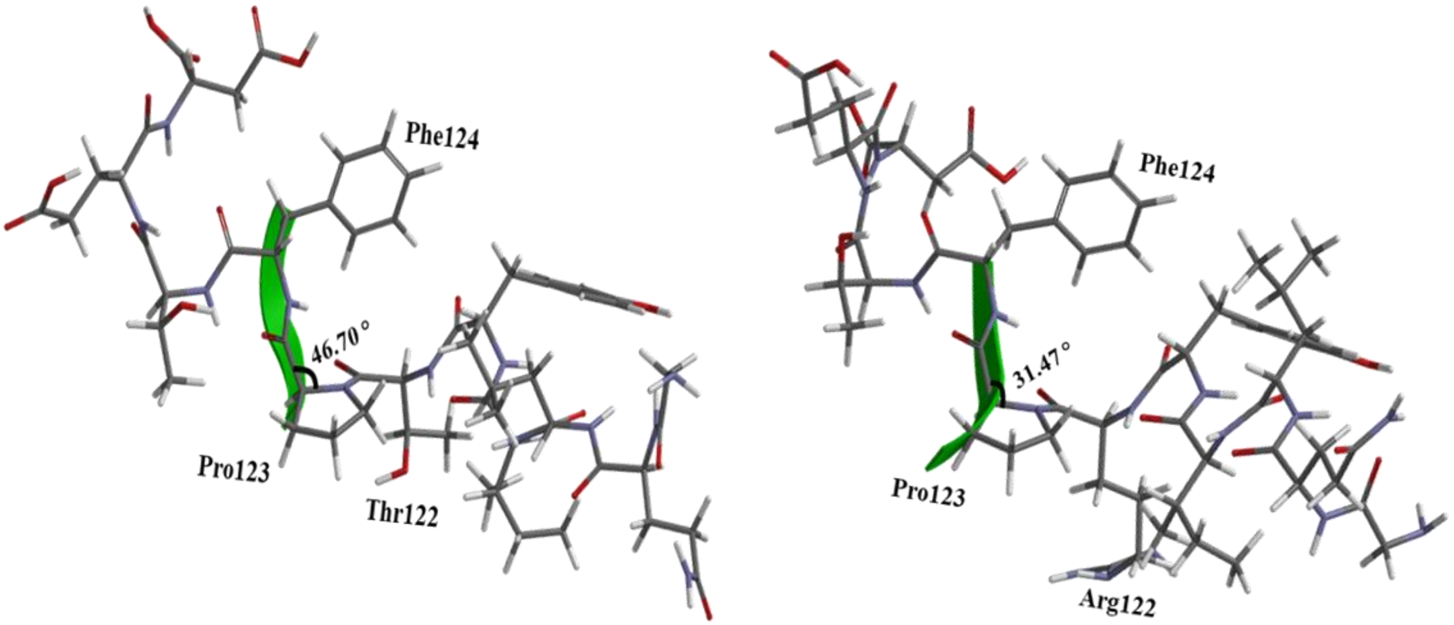

Sup.Figure 3. *Angle change for PS2 wild type protein (Left) and Thr122Arg mutation (Right).*

Table Sup.1

Canonical function TM2-TM3-TM5						
**Protein PS2 (239)**	**Energy total (kJ/mol)**	**Amount of Aβ40**	**Amount Aβ42**	**Delta40**	**Delta42**	**Hidropathicity (239)**
Wild type 1	328534,4815	1,00	1,00	0,00	0,00	2,644
M239I	331629,757	0,44	3,14	-0,56	2,14	2,933
M239V	330776,1052	0,31	3,30	-0,69	2,30	2,900

A

Moonlighting function TM1-TM2							
**Protein PS2 (130)**	**Energy total (kJ/mol)**	**Amount of Aβ40**	**Amount Aβ42**	**Delta40**	**Delta42**	**Phosphorylation (130)**	**Hidropathicity (130)**
Wild type 2	334625,8934	1,00	1,00	0,00	0,00	0,627	-1,589
S130L	335897,732	0,86	1,22	-0,14	0,22	0	-1,078

B

Moonlighting function TM1-TM2						
**Protein PS2**	**Energy total (kJ/mol)**	**Amount of Aβ40**	**Amount Aβ42**	**Phosphorylation (122)**	**Phosphorylation (125)**	**Hidropathicity (122)**
Wild type	333370,1626	N/D	N/D	0,513	0,431	-0,022
T122R	318579,7143	N/D	N/D	0	0,712	-0,444

C

Table Sup.1 *Energetic values calculated in the system with PS2 in the γ-secretase enzyme: A) Energy average of canonical PS2 function and M239I, M239V mutations; B) Energy average and characterization for the wild type and mutated moonlighting function Ser130Leu; C) Energy average and characterization for the moonlighting wild type and mutated Thr122Arg function.*
